# Real-World Clinical Outcomes and Treatment Patterns in Patients with Unresectable Stage III NSCLC Treated with CCRT With or Without Durvalumab as Consolidation Therapy: A Retrospective Cohort Study

**DOI:** 10.3390/jcm15218561

**Published:** 2026-11-03

**Authors:** Pragya Rai, Yu-Han Kao, Xiaohan Hu, Ashwini Arunachalam, Andrew Song, Stephen V. Liu

**Affiliations:** 1 Merck & Co., Inc., Rahway, NJ 07065, USA; yu-han.kao@merck.com (Y.-H.K.); xiaohan.hu@merck.com (X.H.); ashwini.arunachalam@merck.com (A.A.); andrew.song@merck.com (A.S.); 2 Lombardi Comprehensive Cancer Center, Georgetown University, Washington, DC 20007, USA; stephen.liu@gunet.georgetown.edu

**Keywords:** non-small cell lung cancer, disease progression, treatment outcomes, overall survival

## Abstract

**Background:** Durvalumab is currently approved as consolidation therapy for patients with unresectable stage III non-small cell lung cancer (NSCLC) without disease progression following concurrent chemoradiotherapy (cCRT). This study described real-world characteristics, disease progression, subsequent treatment, and clinical outcomes according to durvalumab receipt. **Methods:** This retrospective cohort study used the N-Power Medicine database. Adults diagnosed with unresectable stage III NSCLC who initiated cCRT between February 2018 and October 2023 and subsequently received durvalumab or no durvalumab and were followed until October 2025. Baseline characteristics, disease progression, and subsequent treatment patterns were summarized descriptively. Kaplan–Meier methods were used to estimate overall survival (OS) and time to death or distant metastasis (TDDM). **Results:** Among 717 patients who initiated cCRT, 487 received cCRT followed by durvalumab and 230 received cCRT only. Baseline characteristics were broadly similar between cohorts. Median theoretical follow-up was 60.5 and 60.8 months, respectively. Disease progression was observed in both groups, with higher rates in the cCRT-only cohort (48.7% vs. 40.9%). TDDM was 26.7 months in the cCRT + durvalumab cohort and 5.6 months in the cCRT-only cohort. Median OS was 42.6 months and 11.4 months, and OS events occurred in 55.2% and 80.0% of patients, respectively. In the cCRT + durvalumab cohort, OS rates at 12, 36 and 60 months were 83.6%, 56.0%, and 42.8%. **Conclusions:** In real-world practice, approximately one-third of patients with unresectable stage III NSCLC did not receive durvalumab following cCRT. Despite consolidation therapy, a substantial proportion of patients continue to experience disease progression, highlighting ongoing unmet need in this population.

## 1. Introduction

Lung cancer is the leading cause of cancer-related deaths in the United States (US), accounting for approximately 124,990 deaths annually [1]. Non-small cell lung cancer (NSCLC) accounts for >80% of lung cancer cases, and 20–35% of patients present with stage III (American Joint Committee on Cancer Staging Manual [AJCC] 9th Edition) locally advanced disease [2,3]. Platinum-based concurrent chemoradiotherapy (cCRT) followed by surveillance became the standard of care for unresectable stage III NSCLC in the early 2000s, and remained so until 2018 [4]. Despite the curative intent of cCRT, relapse rates were high and approximately 50% of recipients developed distant metastases [1]. Moreover, median progression-free survival (PFS) was approximately 8 months and 5-year overall survival (OS) was approximately 37% [5,6]. These outcomes highlighted the need for alternative therapies that reduce recurrence and improve long-term survival outcomes in patients with unresectable stage III NSCLC [5,6].

This led to the approval of durvalumab, an anti-programmed death ligand (PD-L)-1 inhibitor, by the US Food and Drug Administration (FDA) as consolidation therapy for patients with unresectable stage III NSCLC whose disease had not progressed following cCRT based on results from the PACIFIC trial in February 2018 [7]. Since approval, durvalumab has been established as the standard of care (SOC) for unresectable stage III NSCLC [7,8,9]. Despite these advances, approximately 67% of patients receiving durvalumab experience disease progression within five years, suggesting durable disease control remains a key concern for many patients administered with this therapy [7,10]. Additionally, a notable proportion of patients are ineligible for durvalumab due to residual toxicity from cCRT, poor performance status, or early progression following cCRT [11,12,13]. This underscores the need to explore strategies to improve the long-term outcomes and durability of disease control in patients with unresectable stage III NSCLC.

Although several real-world studies have evaluated cCRT followed by durvalumab in different settings, evidence is evolving, particularly regarding long-term outcomes and treatment patterns in diverse patient populations and community settings [9,10,11,14]. This study provides the most recent data with extended follow-up, offering insights into the real-world utilization of cCRT followed by durvalumab in clinical practice settings [15]. Specifically, this study aimed to describe patient and clinical characteristics based on durvalumab receipt, characterize progression status and subsequent treatment patterns, and assess real-world clinical outcomes in patients with unresectable stage III NSCLC treated with cCRT followed by with or without durvalumab after the establishment of durvalumab as SOC in this patient population [3].

## 2. Materials and Methods

### 2.1. Study Population

This retrospective cohort study used data from the N-Power Medicine (NPM) analytical data set (ADS) Enriched Cohort, a subset of all lung cancer patients in the NPM database with enriched data, manually abstracted from patient charts by NPM oncology data specialists. The NPM database integrates US electronic health records, data warehouses, cancer registries, medication administration records, and order entry systems, enabling comprehensive capture of patient characteristics, staging, treatments, and recurrence using standardized criteria.

Informed consent for participation is not required as the study involved only the secondary use of data that were de-identified in compliance with the Health Insurance Portability and Accountability Act, specifically, 45 CFR § 164.514.

Patients were screened for inclusion if they were diagnosed with NSCLC during the study identification period: February 2018 to October 2023. Observation continued until data cutoff of October 2025, death, or loss to follow-up. Patients were included if (1) they were adults (≥18 years) with histologically confirmed NSCLC, (2) who had stage III (IIIA, IIIB, or IIIC) disease per AJCC 8th edition, (3) with no prior lung cancer surgery, and (4) initiated cCRT, defined as receipt of chemotherapy and radiation therapy within 14 days of each other. Patients were excluded if they had prior primary cancer before NSCLC diagnosis, prior surgery, radiation, or systemic therapy outside of the cCRT window, or had participated in interventional clinical trials.

For the analysis, the index date was defined as the date of initiation of cCRT (unless otherwise specified).

### 2.2. Endpoint Definitions

Treatment patterns examined concurrent chemotherapy, duration of cCRT and durvalumab therapy, and first subsequent treatment following disease progression, for both overall and stratified by duration of durvalumab. Disease progression was considered if there was a record of loco-regional recurrence or distant metastasis; in addition, initiation of subsequent systemic anticancer therapy following completion or discontinuation of cCRT or durvalumab, in the absence of recorded loco-regional recurrence or metastasis, was considered indicative of disease progression. Time to death or distant metastasis (TDDM) was defined as the time from the initiation of treatment of interest to the earlier date of distant metastasis or death. Patients without any of these event indicators were censored at either last abstraction date or other primary cancer diagnosis or data-cutoff date. OS was defined as the time from index date to date of death. Patients without a death indicator were censored at the data cutoff date. Theoretical follow-up was defined by the data-cutoff date.

Exploratory analysis of OS was also conducted in a subset of patients from the timepoint of initiation of durvalumab to death or data cutoff date (whichever event occurred first). In addition, clinical outcomes were further stratified by histology (non-squamous and squamous cell carcinoma) and PD-L1 expression levels (≥1% and <1%).

### 2.3. Statistical Analysis

Patient and treatment characteristics, disease progression status, and subsequent treatment patterns were assessed descriptively. Kaplan–Meier analyses were applied for estimating TDDM and OS. Baseline characteristics, first subsequent treatment, and OS analysis were presented by histology and PD-L1 status. No formal testing was conducted between treatment groups. All analyses were performed using R version 4.5.2.

## 3. Results

### 3.1. Patient Population

**Figure 1:**
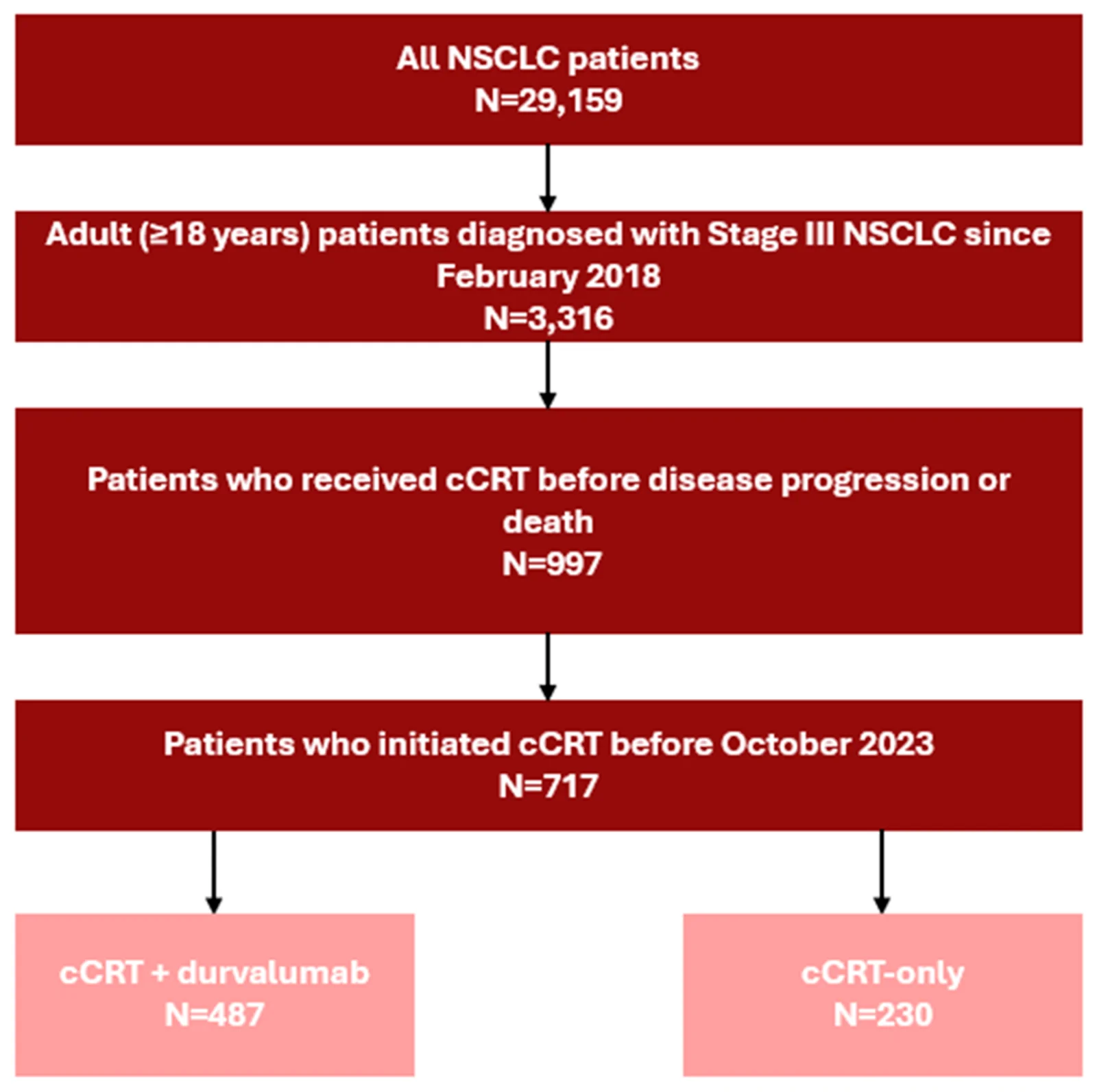
Patient attrition flow chart. cCRT: concurrent chemoradiotherapy; NSCLC: non-small cell lung cancer.

A total of 29,159 patients with NSCLC were enrolled in the NPM database at time of study commencement, of which 3316 adults were diagnosed with stage III NSCLC since February 2018. Patients who had received cCRT before disease progression or death were identified (N = 997). Further, patients initiating cCRT before October 2023 were included (N = 717). Of those, 67.9% (N = 487) received durvalumab as consolidation therapy following cCRT (cCRT + durvalumab group) while 32.1% (N = 230) did not receive durvalumab (cCRT-only group) (Figure 1). The median theoretical follow-up time was 60.5 months in the cCRT + durvalumab cohort and 60.8 months in the cCRT-only cohort (Table 1).

### 3.2. Baseline Characteristics

Overall, baseline characteristics were broadly similar between the cCRT + durvalumab and cCRT-only cohorts. However, a higher proportion of females (46.9% vs. 39.1%) and patients tested for PD-L1 levels (54.6% vs. 50.4%) were in the cCRT + durvalumab cohort than in the cCRT-only cohort, respectively. The cCRT-only group had a higher proportion of patients with ECOG performance status ≥ 2 (19.2% vs. 12.8%) and more patients with positive EGFR mutation status (8.7% vs. 5.6%, respectively) (Table 1). In addition, the cCRT-only cohort included more patients with squamous cell carcinoma (53.0% vs. 49.5%) and stage IIIC disease (11.3% vs. 8.0%) (Table 1).

### 3.3. Treatment Patterns

The most common concurrent chemotherapy administered in both cohorts was carboplatin + paclitaxel (72.7% in the cCRT + durvalumab cohort and 78.7% in the cCRT-only cohort). In the cCRT + durvalumab cohort, cisplatin + etoposide (9.4%) and carboplatin + pemetrexed (8.6%) were more frequently administered compared to the cCRT-only cohort (5.7% and 6.5%, respectively). The use of other concurrent chemotherapy regimens was similar across both cohorts (9.2% and 9.1%, respectively).

The duration of cCRT was consistent between cohorts, with a median duration of 1.5 months (IQR: 1.4, 1.9 in the cCRT + durvalumab cohort; IQR: 1.4, 2.0 in the cCRT-only cohort). For patients in the cCRT + durvalumab cohort, durvalumab therapy lasted a median of 8.5 months (IQR: 2.8, 11.8), and the median interval between completion of cCRT and initiation of durvalumab was 1.2 months (IQR: 0.9, 1.8).

Overall, 40.9% of patients in the cCRT + durvalumab cohort and 48.7% of patients in the cCRT-only cohort showed evidence of disease progression (Table 2). Distant disease progression was most commonly observed: in 29.4% of patients in the cCRT + durvalumab group and 36.5% of patients in the cCRT-only group. The median time from cCRT initiation to distant metastasis was 9.2 months (range: 2.4, 53.2) with cCRT + durvalumab and 4.8 months (range: 0.1, 55.7) with cCRT-only. Loco-regional disease progression occurred in 3.9% of patients in the cCRT + durvalumab cohort, and in 3.0% of patients in the cCRT-only cohort.

The median time from cCRT initiation to first subsequent treatment was 12.7 months in the cCRT + durvalumab cohort and 5.4 months in the cCRT-only cohort (Table 2). Immunotherapy was the most common first subsequent treatment in both cohorts (20.4% in the cCRT + durvalumab cohort and 39.6% in the cCRT-only cohort), followed by chemotherapy (13.6% and 5.5%) and targeted therapy (9.3% and 4.4%) (Table 2). First subsequent treatment stratified by type of disease progression is presented in Supplementary Figure S1.

Within the cCRT + durvalumab cohort, immunotherapy remained the most common first subsequent treatment among patients who received durvalumab for <3 months (22.0%) and 6–<12 months (26.8%). In contrast, among those treated with durvalumab for 3–<6 months, a high proportion of patients (22.6%) received chemotherapy as first subsequent treatment (Table 3).

### 3.4. Clinical Outcomes

**Figure 2:**
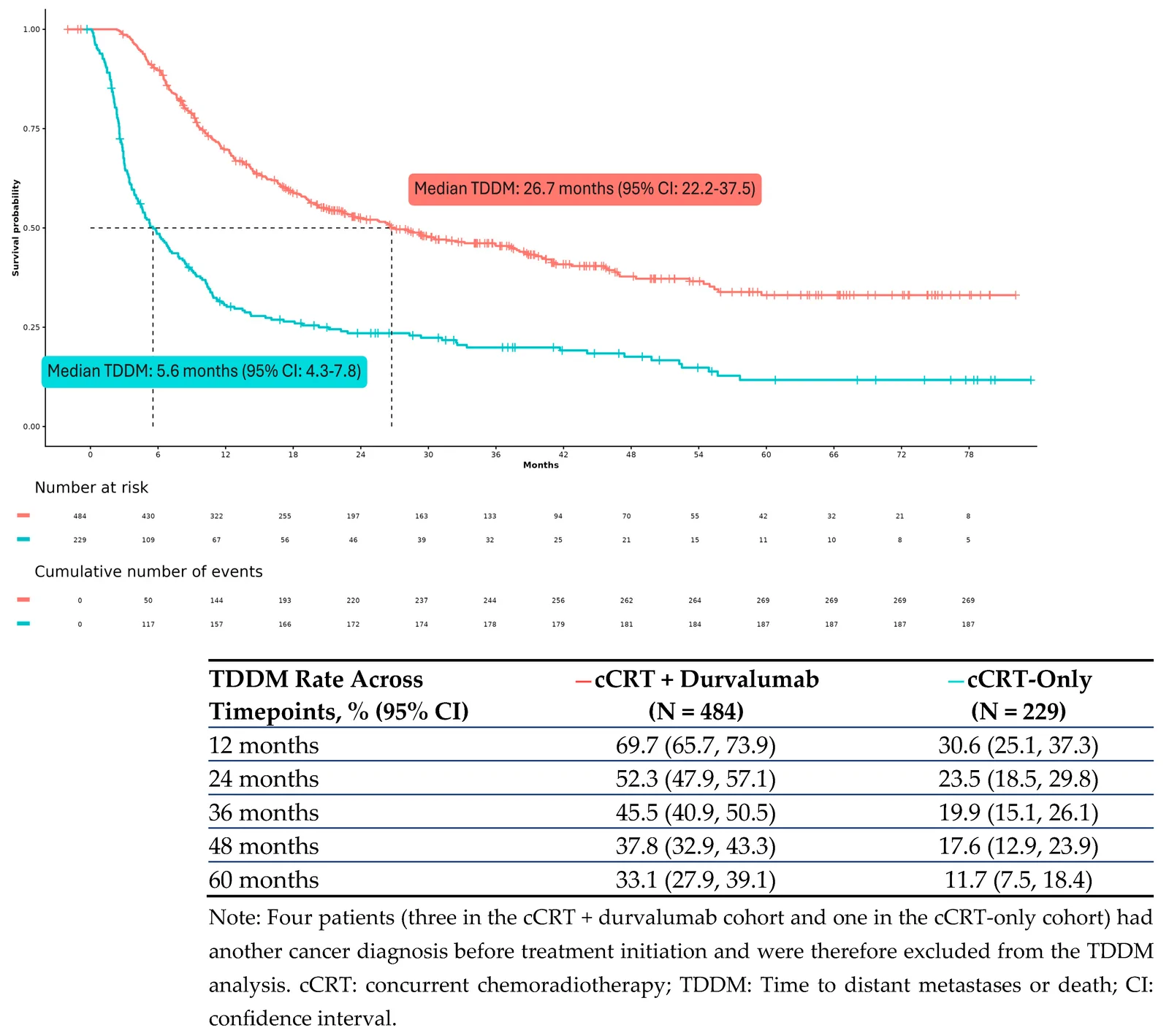
TDDM from cCRT initiation. **—** cCRT + durvalumab **—** cCRT-only.

The median TDDM was 26.7 months (95% CI: 22.2, 37.5) in the cCRT + durvalumab cohort and 5.6 months (95% CI: 4.3, 7.8) in the cCRT-only cohort (Figure 2).

**Figure 3:**
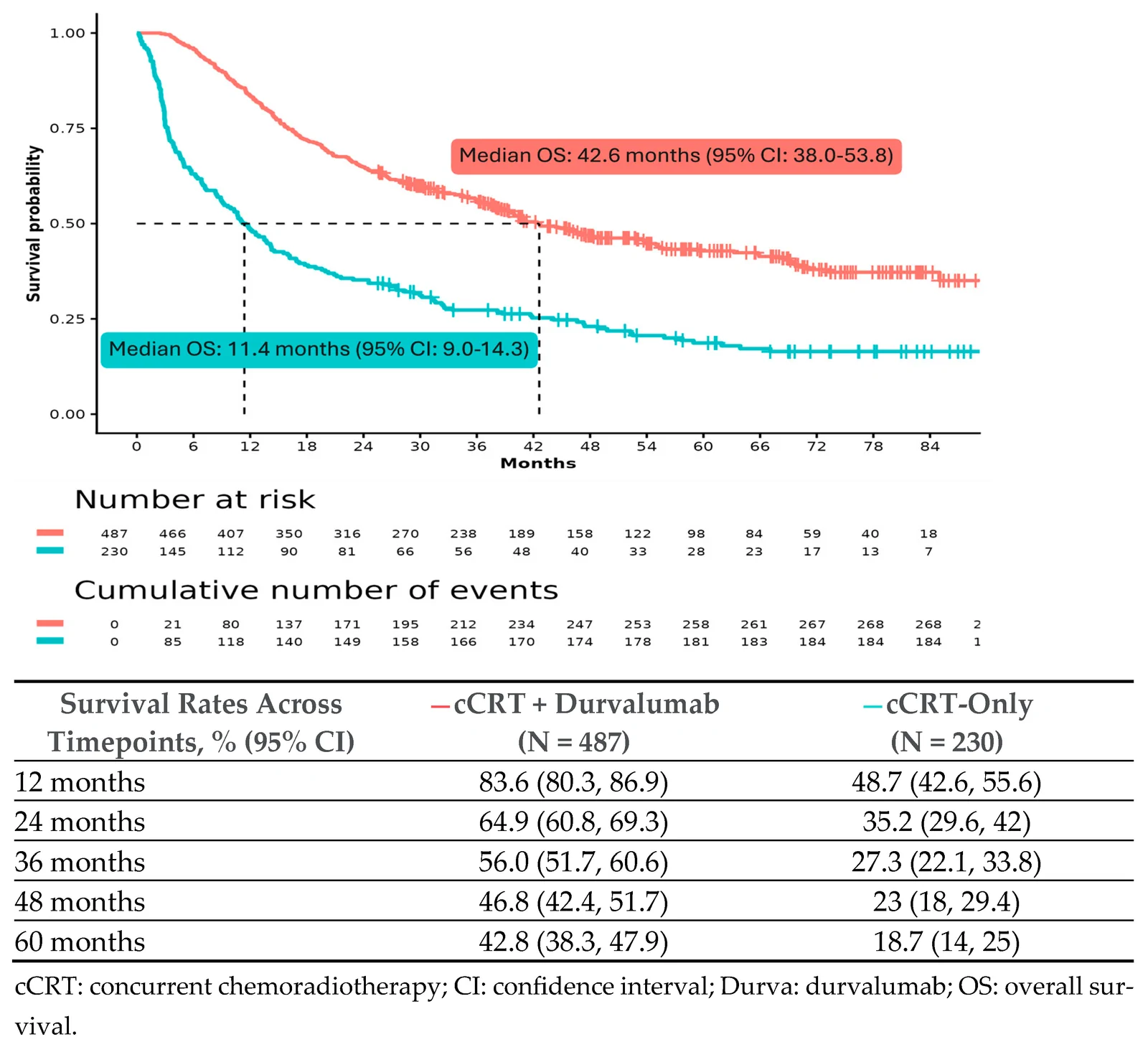
Overall survival from cCRT initiation. **—** cCRT + durvalumab **—** cCRT-only.

In total, 55.2% of patients in the cCRT + durvalumab cohort and 80.0% of patients in the cCRT-only cohort experienced an OS event by the time of data cut-off. The median OS was 42.6 months (95% CI: 38.0–53.8) for patients receiving cCRT + durvalumab and 11.4 months (95% CI: 9.0–14.3) for those treated with cCRT-only. Survival rates were 83.6%, 56.0% and 42.8% at 12, 36 and 60 months, respectively, in the cCRT + durvalumab cohort, and 48.7%, 27.3% and 18.7%, respectively, in the cCRT-only cohort (Figure 3).

### 3.5. Exploratory Analysis

#### 3.5.1. Overall Survival from Initiation of Durvalumab

In patients who received cCRT + durvalumab, median OS from initiation of durvalumab was 39.3 months (95% CI: 34.8–50.8). Survival rates from durvalumab initiation were 76.2% at 12 months, 53.5% at 36 months, and 42.9% at 60 months (Supplementary Figure S2).

##### Outcomes by Histology

Baseline characteristics stratified by histology are presented in Supplementary Table S1. Baseline characteristics were broadly similar between the overall cCRT + durvalumab group and histology-specific subgroups in this cohort; however, a higher proportion of patients with squamous histology had early nodal involvement (N0-N1).

Among patients in the cCRT + durvalumab cohort who experienced disease progression, immunotherapy remained the most common first subsequent treatment across histology (45.9–51.7%). Chemotherapy was also frequently used (29.7–34.5%), while targeted therapy use was comparatively low (13.7–24.3%). Among patients in the cCRT-only cohort who experienced disease progression, immunotherapy similarly dominated as the first subsequent treatment across histology (81.0%). Targeted therapy was used in 9.5% to 14.3% of patients, while chemotherapy was used in 4.8% to 9.5% of patients (Supplementary Figure S3).

In the cCRT + durvalumab cohort, median OS was 59.6 months (95% CI: 45.6–NA) in patients with non-squamous histology and 38.0 months (95% CI: 30.3–45.9) in patients with squamous histology. In the cCRT-only cohort, median OS was 13.4 months (95% CI: 8.9–27.5) in patients with non-squamous histology and 10.8 months (95% CI: 6.8–14.2) in patients with squamous histology. Survival rates stratified by histology are presented in Supplementary Figure S4.

When indexed from the initiation of durvalumab, median OS was 55.8 months (95% CI: 41.9–NA) in patients with non-squamous histology and 34.6 months (95% CI: 27.1–41.7) in those with squamous histology (Supplementary Figure S5).

##### Outcomes Stratified by PD-L1 Status

Baseline characteristics stratified by PD-L1 status are presented in Supplementary Table S2. In the cCRT + durvalumab cohort, more patients with PD-L1 ≥ 1% had Charlson Comorbidity Index ≥ 1 compared to those with PD-L1 < 1% (46.9% vs. 39.4%). In the cCRT-only cohort, more patients with PD-L1 ≥1% had stage IIIB disease than patients with PD-L1 < 1% (53.7% vs. 46.2%).

In the cCRT + durvalumab cohort, median TDDM was 37.5 months (95% CI: 28.1, 55.8) in patients with PD-L1 ≥1% and 16.7 months (95% CI: 12.6, NA) in patients with PD-L1 < 1%. In the cCRT-only cohort, median TDDM was 6.6 months (95% CI: 4.5, 10.3) and 3.9 months (95% CI: 2.9, 9.9), respectively (Supplementary Figure S6).

In the cCRT + durvalumab cohort, 50% of patients with PD-L1 < 1% and 36.8% of patients with PD-L1 ≥ 1% experienced disease progression. In the cCRT-only cohort, 46.2% of patients with PD-L1 < 1% and 52.2% of patients with PD-L1 ≥ 1% experienced disease progression (Supplementary Table S3).

The distribution of first subsequent treatment varied across PD-L1 subgroups and initial treatment cohorts. In the cCRT + durvalumab cohort, patients with PD-L1 ≥ 1% most frequently received immunotherapy as first subsequent treatment (56.5%), followed by chemotherapy (21.7%) and targeted therapy (21.7%), whereas among PD-L1 < 1% patients, chemotherapy was predominant (62.5%), with lower use of targeted therapy (25.0%) and immunotherapy (12.5%). In the cCRT-only cohort, immunotherapy was the most common first subsequent treatment in both PD-L1 ≥ 1% (72.7%) and PD-L1 < 1% (83.3%) patients, with smaller proportions receiving targeted therapy (18.2% and 16.7%, respectively). In the cCRT-only cohort, chemotherapy was infrequently used in PD-L1 ≥ 1% patients (9.1%) and not used in PD-L1 < 1% patients (Supplementary Figure S7).

When indexed from the initiation of cCRT, median OS was 55.0 months (95% CI: 42.4–NA) in patients with PD-L1 ≥ 1% and 65.9 months (95% CI: 17.3–NA) in patients with PD-L1 < 1% in the cCRT + durvalumab cohort. In the cCRT-only cohort, median OS was 12.0 months (95% CI: 8.5–44.1) in patients with PD-L1 ≥ 1% and 9.1 months (95% CI: 3.9–16.9) in patients with PD-L1 < 1 (Supplementary Figure S8).

When indexed from durvalumab initiation, median OS from the date of durvalumab initiation was 52.5 months (95% CI: 38.8–NA) in patients with PD-L1 ≥ 1% and 62.8 months (95% CI: 14.6–NA) in patients with PD-L1 < 1% (Supplementary Figure S9).

## 4. Discussion

Durvalumab consolidation therapy following cCRT is the current SOC for unresectable stage III NSCLC, and findings from this study support its real-world clinical benefit compared with cCRT alone. Patients treated with cCRT + durvalumab achieved longer median OS from cCRT initiation (42.6 months vs. 11.4 months) and maintained higher OS rates at all time points up to five years compared to the cCRT-only cohort. These results are consistent with prior real-world study, which reported that patients treated with durvalumab as consolidation therapy had a median OS of five years, versus a median OS of 1.75 years for patients receiving cCRT only [16]. In addition, results from this study are consistent with the five-year outcomes from the PACIFIC trial, which demonstrated a median OS of 47.5 months with the administration of cCRT + durvalumab versus 29.1 months with cCRT-only [17]. Furthermore, when indexed from durvalumab initiation, median OS was favorable (39.3 months) and early survival was high (76.2% at 12 months, and 62.0% at 24 months) in the cCRT + durvalumab cohort. These findings are similar to those from the PACIFIC and PACIFIC-R studies, which reported OS rates of 66.3% and 71.2%, respectively, at 24 months for patients administered with durvalumab [12,14,18]. Beyond survival benefit, the cCRT + durvalumab cohort had reduced disease progression risk compared with cCRT-only, as shown by a higher proportion of patients without disease progression (59.1% vs. 51.3%), fewer distant metastases (29.4% vs. 36.5%), and longer time to subsequent treatment (median 12.7 vs. 5.4 months). Additionally, the median TDDM in the cCRT + durvalumab cohort was 26.7 months, whereas the median TDDM in the cCRT-only cohort was 5.6 months, indicating potential delayed metastasis associated with durvalumab. These findings align with findings from the PACIFIC trial, where durvalumab significantly prolonged TDDM (23.2 vs. 14.6 months) compared with placebo. In this trial, durvalumab also demonstrated an improved OS rate at five years (42.9% vs. 33.4%) [7,17].

Despite these improvements, sustained disease control remains an unmet need with durvalumab. In this study, 40.9% of patients in the cCRT + durvalumab cohort experienced disease progression, with the majority (71.8%) developing distant metastases. Importantly, 43.2% went on to receive subsequent systemic therapy. This is supported by broader real-world studies, which found that 52.7% patients experienced disease progression (at the time of the study database cut-off) and a substantial proportion of patients required subsequent systemic therapy following durvalumab receipt [12,19]. The need for continued treatment suggests that current consolidation strategies, despite offering survival benefits, do not achieve long-term disease control for all patients, highlighting an unmet need for more effective therapeutic options which support patients to achieve long-term disease control without the need for subsequent treatment [20].

These findings also address a common clinical question on whether patients previously treated with immunotherapy in earlier stages receive immunotherapy in later stages [21]. The results showed that in the cCRT + durvalumab cohort, 46.3% of patients who were previously treated with durvalumab received immunotherapy in subsequent lines of therapy. Immunotherapy use as the first subsequent treatment in patients who experienced distant disease progression in the cCRT-only cohort was higher (78.6%), although this is expected as patients who were previously treated with immunotherapy, but failed treatment are less likely to initiate subsequent immunotherapy treatment. Nonetheless, these results indicate that prior immunotherapy exposure does not preclude subsequent immunotherapy use in practice.

Within this study, some important differences in outcomes were observed, depending on patient histology and biomarker status. Specifically, histology and biomarker status shaped OS outcomes when indexed from durvalumab initiation. Patients with squamous cell carcinoma had a shorter median OS (34.6 months) compared to patients with non-squamous cell carcinoma (55.8 months). In addition, OS rate at five years was lower for patients with squamous cell carcinoma (37.3%), compared to patients with non-squamous cell carcinoma (49.8%), consistent with historically poorer outcomes observed in this subgroup [22].

The results show that across the majority of timepoints, patients with PD-L1 < 1% had lower OS rate than patients with PD-L1 ≥1%, suggesting that patients with PD-L1 < 1% who are administered with durvalumab as consolidation therapy may have poorer outcomes compared to those with PD-L1 ≥1%. These findings align with those from the PACIFIC study, which reported that out of 63.3% of patients who had available data for PD-L1 expression, a higher proportion of patients treated with durvalumab in the PD-L1 ≤ 1% subgroup experienced an event compared to the PD-L1 ≥ 1% subgroup [18]. These findings suggest that long-term disease control with durvalumab remains an unmet need for patients with squamous cell carcinoma, and with low PD-L1 expression, reinforcing the need for treatment strategies which provide better outcomes in these populations.

Notably, the results of this study, and that of previous findings, are aligned with the expectation that durvalumab is more effective in patients with high PD-L1 expression given its mechanism of action as a PD-L1 inhibitor. However, patients with PD-L1 < 1% still demonstrated improved median OS outcomes with durvalumab compared to cCRT-only, despite the reduced magnitude of benefit relative to patients who express PD-L1 ≥1%. A potential explanation is that chemoradiotherapy has been shown to upregulate PD-L1 expression, potentially making tumors more susceptible to PD-L1 inhibitors regardless of baseline status [23]. PD-L1 expression is also subject to tumor heterogeneity, meaning baseline PD-L1 may not fully capture the immune landscape at the time of consolidation therapy [24].

When interpreting PD-L1–stratified outcomes, it is important to consider study design characteristics that may have influenced the observed OS estimates, particularly in the PD-L1 < 1% subgroup. In the cCRT + durvalumab group, the PD-L1 < 1% subgroup was small (*n* = 40) compared to the PD-L1 ≥1% subgroup (*n* = 174) which could have resulted in unstable Kaplan–Meier estimates, wide confidence intervals, and non-estimable median survival bounds. Additionally, in the cCRT + durvalumab cohort, there was heavy censoring in the PD-L1 <1% subgroup, with 16 out of 20 events occurring within 18 months, resulting in a shorter observed median follow up duration in the PD-L1 <1% subgroup (29.2 months, IQR: 13.7–50.5 months), compared to the PD-L1 ≥ 1% subgroup (37.2 months, IQR: 23.2–53.0 months). This early event clustering may have contributed to an inflation of the median OS estimate of 65.9 months in the PD-L1 < 1% subgroup, and an overestimation of OS rates in the later portion of the Kaplan–Meier curve. Despite higher median OS in this subgroup, the median TDDM was shorter in the cCRT + durvalumab cohort for patients with PD-L1 < 1% (16.7 months) compared to PD-L1 ≥ 1% (37.5 months), in line with what is expected of durvalumab treated patients. Notably, when examining OS rates at 12 and 36 months, the cCRT + durvalumab PD-L1 ≥ 1% subgroup demonstrated favorable outcomes (88.5% and 62.6%, respectively) compared to the cCRT + durvalumab PD-L1 < 1% subgroup (80.0% and 55%, respectively). Furthermore, both durvalumab-treated subgroups showed markedly improved OS rates compared to cCRT-only PD-L1 < 1% patients (46.2% and 19.2% at 12 and 36 months, respectively, in the cCRT-only PD-L1 < 1% subgroup), supporting the expected biological mechanism of PD-L1 inhibition. Lastly, in the cCRT + durvalumab cohort, the lower bound of the 95% CI for median OS in the PD-L1 < 1% subgroup was substantially lower than that in the PD-L1 ≥ 1% subgroup (17.3 months versus 42.2 months), indicating uncertainty in this estimate. Therefore, survival results for PD-L1 <1% expressors should be interpreted with caution.

Beyond observed efficacy outcomes, broader real-world challenges also contribute to unmet need when considering durvalumab use. In this study, approximately one-third of the patients with unresectable stage III NSCLC did not receive durvalumab following cCRT. However, this may reflect appropriate clinical decision-making in line with approved treatment guidelines as patients with early disease progression post-cCRT and those with EGFR mutations, who may be directed toward alternative therapies, likely account for a notable proportion of this group. This is supported by broader research which found that 31–48% of eligible patients in real-world practice either did not initiate durvalumab or discontinued early due to progression, comorbidities, or toxicity, underscoring variability in treatment delivery and the need for optimized care pathways [25,26]. In addition, a study by Arunachalam et al. reported that 51.5% of patients discontinued durvalumab, primarily due to adverse events (35.8%) and disease progression (28.4%) [15]. Real-world studies further highlight discontinuation driven by immune-related toxicity, particularly pneumonitis, which led to treatment cessation in up to 9.5% of patients who were receiving durvalumab [11,12,25]. Further research is warranted to explore possible reasons that patients do not receive durvalumab following cCRT.

Despite durvalumab consolidation following cCRT representing current SOC, many patients still experience disease progression, highlighting a persistent unmet need. Consideration is needed on treatment options that could improve survival outcomes for patients with unresectable stage III NSCLC from induction. The ongoing phase III KEYLYNK-012 study evaluates the combination of pembrolizumab with cCRT followed by pembrolizumab with or without olaparib versus cCRT followed by durvalumab for the treatment of unresectable, locally advanced, stage III NSCLC [20]. Results from this and similar investigations will be critical in determining whether novel treatment strategies can address the limitations of current durvalumab-based regimens and improve long-term outcomes for patients who remain at risk of disease progression.

The findings of this study must be interpreted in light of its limitations due to the retrospective nature of this study. However, patients in this study were selected randomly based on the inclusion/exclusion criteria to limit selection bias. First, the NPM database represents treatment provided at US community practices. Treatment patterns in these practices may not be representative of all US cancer clinics where NSCLC patients are treated and are not generalizable to the wider US population. Second, immortal time bias may have been introduced in the time-to-event analysis for the cCRT + durvalumab group, as patients would have to be alive at least till the initiation of durvalumab. In our study, the median duration between end of cCRT and initiation of durvalumab was 1.2 months and hence, would not contribute substantially to the longer clinical outcomes observed in this group. Moreover, this cohort is in line with the prescribed label for durvalumab and allowed for comparison with the PACIFIC trial results. Third, disease progression was identified using a combination of chart-documented evidence of loco-regional disease progression or distant metastasis and initiation of subsequent systemic anticancer therapy following completion or discontinuation of cCRT or durvalumab. While initiation of subsequent therapy represents a clinically meaningful and well-documented treatment decision, progression dates and radiographic assessments were not uniformly captured for all patients, which may introduce residual confounding and uncertainty in the precise timing and classification of progression events. Lastly, a formal calculation of sample size and statistical power was not performed due to the descriptive nature of the study.

## 5. Conclusions

Within this study, one-third of patients with unresectable stage III NSCLC did not receive durvalumab following cCRT, warranting future studies to understand the reasons. Additionally, despite the receipt of durvalumab, 40.9% of patients experienced progression. Most patients were re-treated with immunotherapy, accounting for most of the re-treatment overall (46.3% for distant metastasis and 56.7% for loco-regional recurrence) following disease progression. These results highlight an unmet need, and the requirement for effective definitive treatment strategies to improve the durability of clinical outcomes in patients with unresectable stage III NSCLC.

## Figures and Tables

**Table 1:** Baseline characteristics.

Characteristic	cCRT + Durvalumab (N = 487)	cCRT-Only (N = 230)
**Age at Diagnosis (years)**		
Median (IQR)	67 (61, 74)	69 (63, 74)
Mean (SD)	67 (9)	68 (9)
Sex, *n* (%) *		
Female	228 (46.9)	90 (39.1)
Male	257 (52.9)	140 (60.9)
Other	1 (0.2)	0 (0.0)
Race, *n* (%) *		
Black/African American	84 (17.4)	46 (20.1)
White	387 (80.0)	177 (77.3)
Other	13 (2.7)	6 (2.6)
Ethnicity, *n* (%) *		
Hispanic/Latino	19 (4.1)	6 (2.7)
non-Hispanic/non-Latino	445 (95.9)	216 (97.3)
**Histology subgroup, *n* (%)**		
Non-squamous	214 (43.9)	92 (40.0)
Squamous	241 (49.5)	122 (53.0)
Other	32 (6.6)	16 (7.0)
**Stage subgroup, *n* (%)**		
IIIA	220 (45.2)	96 (41.7)
IIIB	218 (44.8)	101 (43.9)
IIIC	39 (8.0)	26 (11.3)
III (undefined)	10 (2.1)	7 (3.0)
N stage, *n* (%) *		
N0–N1	100 (20.6)	41 (18.0)
≥N2	385 (79.4)	187 (82.0)
Smoking Status, *n* (%) *		
Current	219 (45.1)	105 (45.9)
Former	251 (51.6)	110 (48.0)
Never	16 (3.3)	14 (6.1)
Charlson Comorbidity Index, *n* (%) *		
0	266 (65.8)	115 (64.2)
≥1	138 (34.2)	64 (35.8)
ECOG Performance Status at diagnosis, *n* (%) *		
0–1	320 (87.2)	118 (80.8)
≥ 2	47 (12.8)	28 (19.2)
**PD-L1 test, *n* (%)**		
Yes	266 (54.6)	116 (50.4)
No	221 (45.4)	114 (49.6)
PD-L1 status, *n* (%) *		
≥50%	83 (31.2)	33 (28.4)
1–49%	91 (34.2)	34 (29.3)
<1%	40 (15.0)	26 (22.4)
Unknown	52 (19.75)	23 (19.8)
**EGFR test, *n* (%)**		
Yes	233 (47.8)	103 (44.8)
No	254 (52.2)	127 (55.2)
EGFR status, *n* (%) *		
Positive	13 (5.6)	9 (8.7)
Negative	114 (48.9)	38 (36.9)
Theoretical follow-up time **, months		
Median (IQR)	60.5 (43.8, 76.2)	60.8 (46.5, 78.1)
Mean (SD)	59.8 (19.0)	61.3 (19.1)

* Percentages were calculated based on available data. PD-L1 and EGFR values calculated as a percentage of those who were tested for this biomarker. ** Theoretical follow-up was defined as the time from the initiation of treatment (i.e., index date) to data cutoff (October 2025). cCRT: concurrent chemoradiotherapy; ECOG PS: Eastern Cooperative Oncology Group Performance Status; EGFR: epidermal growth factor receptor; IQR: interquartile range; PD-L1: programmed death-ligand 1; SD: Standard deviation.

**Table 2:** Treatment patterns.

	cCRT + Durvalumab (N = 487)	cCRT-Only (N = 230)
**Concurrent chemotherapy regimen, *n* (%)**		
Carboplatin + paclitaxel	354 (72.7)	181 (78.7)
Cisplatin + etoposide	46 (9.4)	13 (5.7)
Carboplatin + pemetrexed	42 (8.6)	15 (6.5)
Other	45 (9.2)	21 (9.1)
**Duration of cCRT therapy, months**		
Median (IQR)	1.5 (1.4, 1.9)	1.5 (1.4, 2.0)
**Mean (SD)**	1.7 (0.5)	2.3 (7.9)
**Duration of durvalumab therapy, months**		
Median (IQR)	8.5 (2.8, 11.8)	NA (NA, NA)
Mean (SD)	7.9 (5.6)	NA (NA)
**Duration between end of cCRT and initiation of durvalumab, months**		
Median (IQR)	1.2 (0.9, 1.8)	NA (NA, NA)
Mean (SD)	1.5 (1.2)	NA (NA)
**Disease progression, *n* (%)**		
Yes *	199 (40.9)	112 (48.7)
Locoregional recurrence	19 (3.9)	7 (3.0)
Distant metastasis	143 (29.4)	84 (36.5)
Initiated subsequent treatment	37 (7.6)	21 (9.1)
**Time from cCRT initiation to distant metastasis, months**		
Median (Range)	9.2 (2.4, 53.2)	4.76 (0.1, 55.7)
Mean (SD)	12.0 (8.9)	7.5 (9.7)
**First subsequent treatment, *n* (%) ^§^**		
Immunotherapy	33 (20.4)	36 (39.6)
Chemotherapy	22 (13.6)	5 (5.5)
Targeted therapy	15 (9.3)	4 (4.4)
**Median time from cCRT initiation to first subsequent treatment (month)**	12.7 (3.8, 56.5)	5.4 (0.3, 56.2)

* Patients who experienced local-regional recurrence or distant metastases, or who initiated subsequent systemic anticancer therapy following completion or discontinuation of cCRT or durvalumab in the absence of recorded loco-regional recurrence or metastasis. ^§^ First subsequent treatment is calculated as a proportion of patients who experienced loco-regional recurrence and distant metastasis in each cohort (cCRT + durvalumab: *n* = 162; cCRT-only: *n* = 91). cCRT: concurrent chemoradiotherapy; EGFR TKI: Epidermal Growth Factor Receptor Tyrosine Kinase Inhibitor; NA: Not applicable; SD: Standard deviation; VEGF: Vascular Endothelial Growth Factor.

**Table 3:** First subsequent treatment in the cCRT + durvalumab cohort, stratified by the duration of durvalumab use.

First Subsequent Treatment, *n* (%)	Overall ^a^ (N = 162)	<3 Months (N = 50)	3–<6 Months (N = 31)	6–<12 Months (N = 56)	≥12 Months (N = 20)
Immunotherapy	33 (20.4)	11 (22.0)	5 (16.1)	15 (26.8)	2 (10.0)
Chemotherapy	22 (13.6)	6 (12.0)	7 (22.6)	7 (12.5)	2 (10.0)
Targeted therapy	15 (9.3)	5 (10.0)	2 (6.4)	5 (8.9)	3 (15.0)

^a^ Few patients (N = 5) did not have details on durvalumab administration and hence were excluded. cCRT: concurrent chemoradiotherapy.

## Data Availability

The data that supports the findings of this study have been originated by N-Power Medicine, Inc. These deidentified data may be made available on request and are subject to a license agreement with N-Power Medicine.

## Supplementary Materials

The following supporting information can be downloaded at: https://www.mdpi.com/article/10.3390/jcm15218561/s1, Table S1: Baseline characteristics stratified by histology; Table S2. Baseline characteristics stratified by PD-L1 status; Table S3: Disease progression, stratified by PD-L1 status; Figure S1 First subsequent treatment following disease progression, stratified by type of disease progression; Figure S2. Survival rate from durvalumab initiation; Figure S3. First subsequent treatment following disease progression, stratified by histology; Figure S4. Overall survival from cCRT initiation, stratified by histology; Figure S5. Overall survival from durvalumab initiation, stratified by histology; Figure S6. TDDM from cCRT initiation, stratified by PD-L1 status; Figure S7. First subsequent treatment following disease progression, stratified by PD-L1 status; Figure S8. Overall survival from cCRT initiation, stratified by PD-L1 status; Figure S9. Overall survival from durvalumab initiation, stratified by PD-L1 status.
