# Supplementary material for: Real-World Clinical Outcomes and Treatment Patterns in Patients with Unresectable Stage III NSCLC Treated with CCRT With or Without Durvalumab as Consolidation Therapy: A Retrospective Cohort Study
Source: J Clin Med. 2026 Nov 3;15(21):8561. doi: 10.3390/jcm15218561 (PMC13763650; doi:10.3390/jcm15218561)
Supplement: Supplementary file 1 [file jcm-15-08561-s001.zip › jcm-4620375-supplementary.pdf]

Supplementary materials

Table S1. Baseline characteristics stratified by histology

Characteristic	cCRT + durvalumab		cCRT-only	
	NSQ (N=214)	SQ (N=241)	NSQ(N=92)	SQ (N=122)
**Age at Diagnosis – Median (Q1, Q3)**	66 (60, 73)	69 (62, 74)	69 (63, 75)	69 (63, 74)
**Sex, n (%)***				
**Female**	121 (56.8)	92 (38.2)	47 (51.1)	34 (27.9)
**Male**	91 (42.7)	149 (61.8)	45 (48.9)	88 (72.1)
**Other**	1 (0.5)	0 (0.0)	0 (0.0)	0 (0.0)
**Race, n (%)***				
**Black/African American**	45 (21.1)	33 (13.8)	21 (22.8)	20 (16.5)
**White**	158 (74.2)	204 (85.0)	69 (75.0)	97 (80.2)
**Other**	10 (4.7)	3 (1.2)	2 (2.2)	4 (3.3)
**Ethnicity, n (%)***				
**Hispanic/Latino**	8 (4.0)	11 (4.7)	5 (5.5)	1 (0.9)
**Non-Hispanic/Non-Latino**	194 (96.0)	223 (95.3)	86 (94.5)	115 (99.1)
**Stage: III, n (%)**	6 (2.8)	3 (1.2)	2 (2.2)	5 (4.1)
**Stage: IIIA**	98 (45.8)	107 (44.4)	39 (42.4)	50 (41.0)
**Stage: IIIB**	96 (44.9)	107 (44.4)	39 (42.4)	55 (45.1)
**Stage: IIIC**	14 (6.5)	24 (10.0)	12 (13.0)	12 (9.8)
**N status, n (%)***				
**N0–1**	26 (12.1)	69 (28.9)	9 (9.9)	28 (23.1)
**≥ N2**	188 (87.9)	170 (71.1)	82 (90.1)	93 (76.9)
**Smoking status, n (%) ***				
**Current**	87 (40.7)	118 (49.2)	38 (41.3)	59 (48.8)
**Former**	118 (55.1)	117 (48.8)	48 (52.2)	56 (46.3)
**Never**	9 (4.2)	5 (2.1)	6 (6.5)	6 (5.0)
**Charlson comorbidity index, n (%)***				

**0**	116 (68.6)	130 (62.5)	43 (64.2)	60 (61.9)
**≥1**	53 (31.4)	78 (37.5)	24 (35.8)	37 (38.1)
**ECOG Status, n (%) ***				
**0–1**	149 (92.5)	149 (82.3)	50 (80.6)	60 (81.1)
**≥2**	12 (7.5)	32 (17.7)	12 (19.4)	14 (18.9)
**PD-L1 test, n (%)**				
**Yes**	117 (54.7)	132 (54.8)	46 (50.0)	63 (51.6)
**No**	97 (45.3)	109 (45.2)	46 (50.0)	59 (48.4)
**PD-L1 status, n (%)**				
**≥50**	42 (35.9)	36 (27.3)	16 (34.8)	14 (22.2)
**1–49**	38 (32.5)	49 (37.1)	10 (21.7)	23 (36.5)
**<1**	15 (12.8)	22 (16.7)	11 (23.9)	15 (23.8)
**Unknown**	22 (18.8)	25 (18.9)	9 (19.6)	11 (17.5)
**EGFR test, n (%)**				
**Yes**	116 (54.2)	104 (43.2)	45 (48.9)	51 (41.8)
**No**	98 (45.8)	137 (56.8)	47 (51.1)	71 (58.2)
**EGFR status, n (%)**				
**Positive**	8 (6.9)	5 (4.8)	6 (13.3)	2 (3.9)
**Negative**	49 (42.2)	58 (55.8)	13 (28.9)	21 (41.1)
**Unknown**	59 (50.9)	41 (39.4)	26 (57.8)	28 (55.0)

*Percentages were calculated based on available data.

PD-L1 and EGFR values calculated as a percentage of those who were tested for this biomarker.
cCRT: concurrent chemoradiotherapy; OS: overall survival; Adeno: Non-squamous cell carcinoma; SCC: Squamous cell carcinoma; ECOG PS: Eastern Cooperative Oncology Group Performance Status; AJCC: American Joint Committee on Cancer; PD-L1: programmed death-ligand 1; EGFR: epidermal growth factor receptor; LN: lymph node

Table S2. Baseline characteristics stratified by PD-L1 status

Characteristic	cCRT + durvalumab		cCRT-only	
	PD-L1 ≥1% (N=174)	PD-L1 <1% (N=40)	PD-L1 ≥1% (N=67)	PD-L1 <1% (N=26)
**Age at Diagnosis – Median (Q1, Q3)**	68 (62, 73)	65 (61, 74)	68 (63, 74)	70 (63, 74)
**Sex, n (%)***				
**Female**	92 (52.9)	19 (47.5)	25 (37.3)	7 (26.9)
**Male**	82 (47.1)	21 (52.5)	42 (62.7)	19 (73.1)
**Other**	0 (0.0)	0 (0.0)	0 (0.0)	0 (0.0)
**Race, n (%)***				
**Black/African American**	30 (17.3)	8 (20.0)	8 (11.9)	8 (30.8)
**White**	138 (79.8)	31 (77.5)	55 (82.1)	17 (65.4)
**Other**	5 (2.9)	1 (2.5)	4 (6.0)	1 (3.8)
**Ethnicity, n (%)***				
**Hispanic/Latino**	8 (4.8)	0 (0.0)	3 (4.5)	2 (7.7)
**Non- Hispanic/Latino**	160 (95.2)	40 (100.0)	63 (95.5)	24 (92.3)
**Histology, n (%)**				
**Non-SCC**	80 (46.0)	15 (37.5)	26 (38.8)	11 (42.3)
**SCC**	85 (48.9)	22 (55.0)	37 (55.2)	15 (57.7)
**Stage: III, n (%)**	0 (0.0)	2 (5.0)	1 (1.5)	0 (0.0)
**Stage: IIIA**	81 (46.6)	16 (40.0)	25 (37.3)	10 (38.5)
**Stage: IIIB**	74 (42.5)	18 (45.0)	36 (53.7)	12 (46.2)
**Stage: IIIC**	19 (10.9)	4 (10.0)	5 (7.5)	4 (15.4)
**LN Involvement, n (%)***				
**N0–1**	41 (23.6)	7 (17.9)	12 (18.2)	3 (11.5)
**≥N2**	133 (76.4)	32 (82.1)	54 (81.8)	23 (88.5)
**Smoking status, n (%)**				
**Current**	74 (42.8)	16 (40.0)	36 (53.7)	12 (46.2)
**Former**	92 (53.2)	24 (60.0)	26 (38.8)	12 (46.2)
**Never**	7 (4.0)	0 (0.0)	5 (7.5)	2 (7.7)

Charlson comorbidity index, n (%)				
**0**	77 (53.1)	20 (60.6)	25 (51.0)	9 (45.0)
**≥1**	68 (46.9)	13 (39.4)	24 (49.0)	11 (55.0)
**ECOG status, n (%)****				
**0–1**	122 (86.5)	27 (90.0)	40 (87.0)	14 (87.5)
**≥2**	19 (13.5)	3 (10.0)	6 (13.0)	2 (12.5)
**PD-L1 test, n (%)**				
**Yes**	174 (100)	40 (100)	67 (100)	26 (100)
**No**	0 (0)	0 (0)	0 (0)	0 (0)
**PD-L1 status, n (%)**				
**50+**	83 (48)	0 (0)	33 (49)	0 (0)
**1–49**	91 (52)	0 (0)	34 (51)	0 (0)
**<1**	0 (0)	40 (100)	0 (0)	26 (100)
**EGFR test, n (%)**				
**Yes**	140 (80)	34 (85)	57 (85)	20 (77)
**No**	34 (20)	6 (15)	10 (15)	6 (23)
**EGFR status, n (%)**				
**Positive**	8 (5.7)	2 (5.9)	4 (7.0)	2 (10.0)
**Negative**	74 (52.9)	19 (55.9)	25 (43.9)	8 (40.0)
**Unknown**	58 (41.4)	13 (38.2)	28 (49.1)	10 (50.0)

*Percentages were calculated based on available data.

** Unknown or missing values on ECOG status are not included in this table.

PD-L1 EGFR values calculated as a percentage of those who were tested for this biomarker.
cCRT: concurrent chemoradiotherapy; OS: overall survival; Adeno: Non-squamous cell carcinoma; SCC: Squamous cell carcinoma; ECOG PS: Eastern Cooperative Oncology Group Performance Status; AJCC: American Joint Committee on Cancer; PD-L1: programmed death-ligand 1; EGFR: epidermal growth factor receptor; LN: lymph node

Figure S1 First subsequent treatment following disease progression, stratified by type of disease progression.

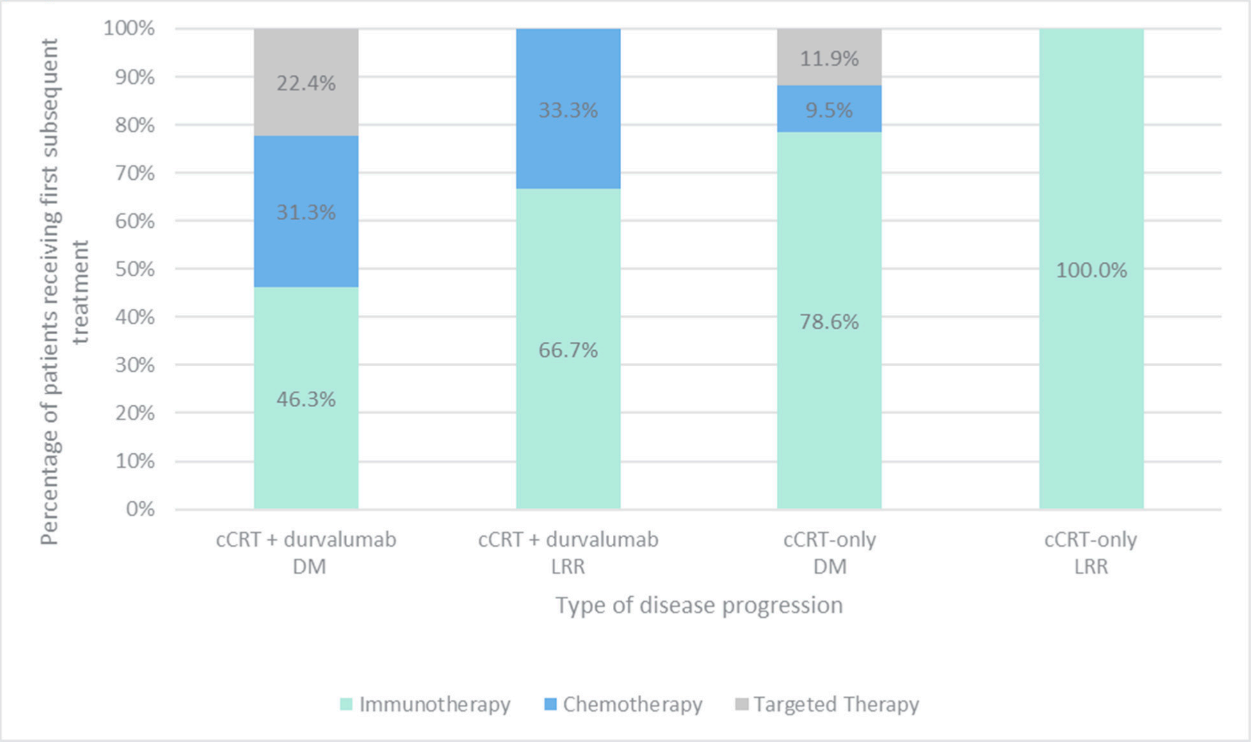

cCRT: Concurrent chemoradiotherapy; DM: Distant metastasis; LRR: Local/regional recurrence

Figure S2. Survival rate from durvalumab initiation

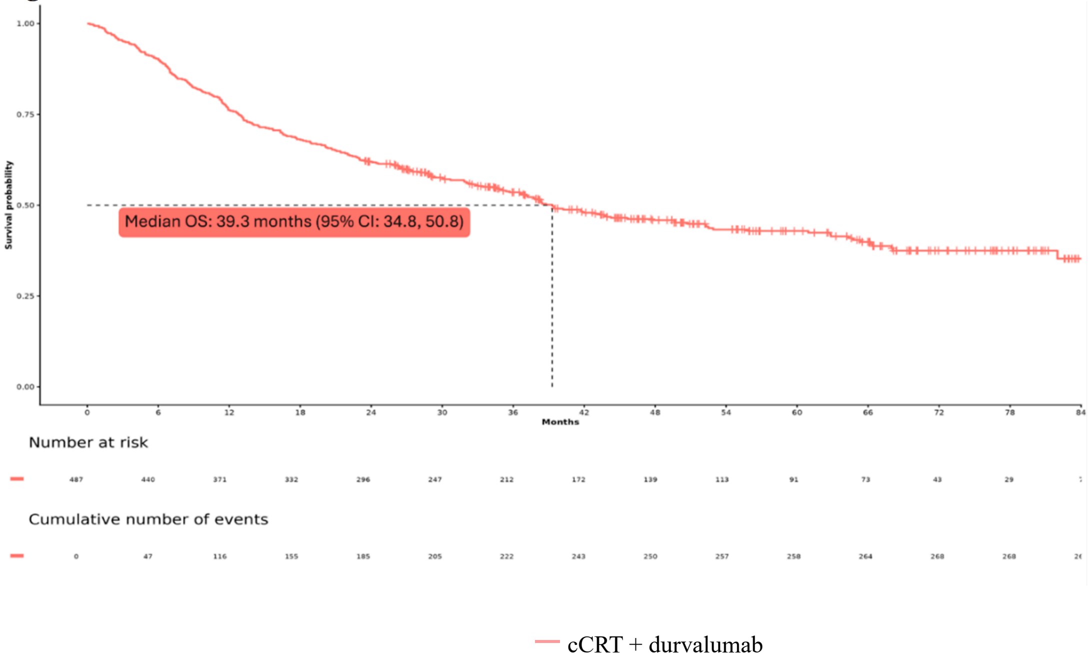

Survival rates across time points, % (95% CI)	cCRT + durvalumab (N=487)
12 months	76.2 (72.5, 80.1)
24 months	62 (57.8, 66.5)
36 months	53.5 (49.2, 58.2)
48 months	45.9 (41.4, 50.8)
60 months	42.9 (38.3, 48)

cCRT: Concurrent chemoradiotherapy; CI: Confidence interval; Durva: durvalumab; OS: overall survival.

Figure S3. First subsequent treatment following disease progression, stratified by histology.

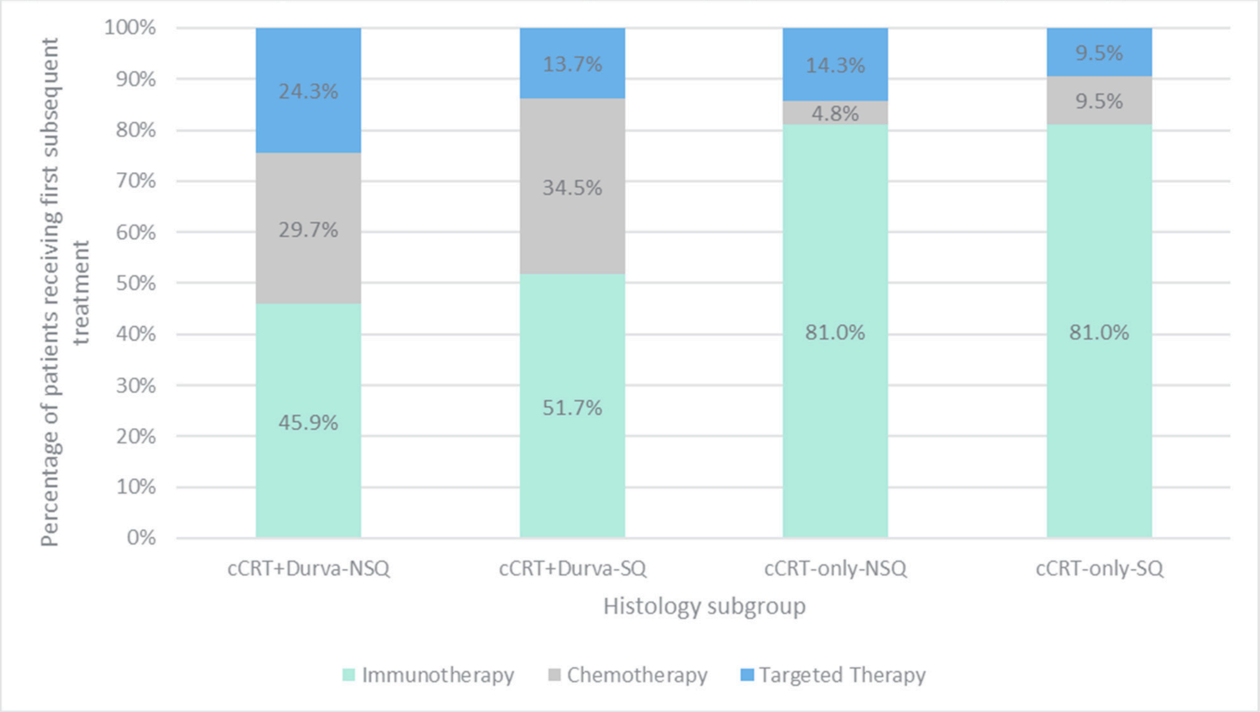

cCRT: Concurrent Chemoradiotherapy; Durva: durvalumab

Figure S4. Overall survival from cCRT initiation, stratified by histology.

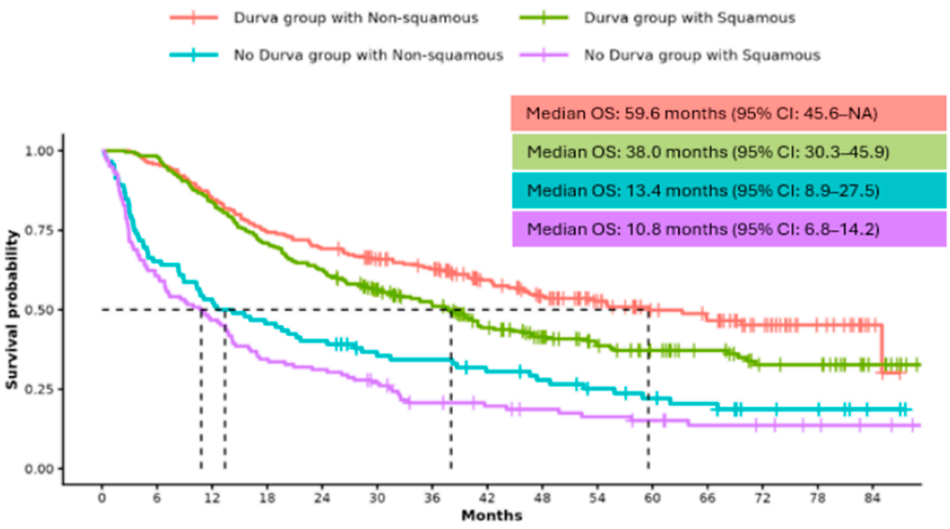

Number at risk

	214	205	182	159	148	132	121	96	80	60	47	42	29	14	4
	241	236	202	171	151	124	105	83	68	53	44	36	25	24	12
	92	60	49	42	37	29	28	24	21	17	14	12	7	6	2
	122	74	57	42	38	31	22	19	16	14	12	9	8	6	4

Cumulative number of events

	0	9	32	55	66	73	79	85	93	95	98	101	102	102	102	1
	0	5	39	70	90	105	115	129	134	138	140	140	144	144	144	1
	0	32	43	50	55	59	60	63	65	67	69	70	71	71	71	[illegible]
	0	48	65	80	84	89	96	97	98	100	101	102	102	102	102	1

cCRT: Concurrent Chemoradiotherapy; Durva: durvalumab

Figure S5. Overall survival from durvalumab initiation, stratified by histology.

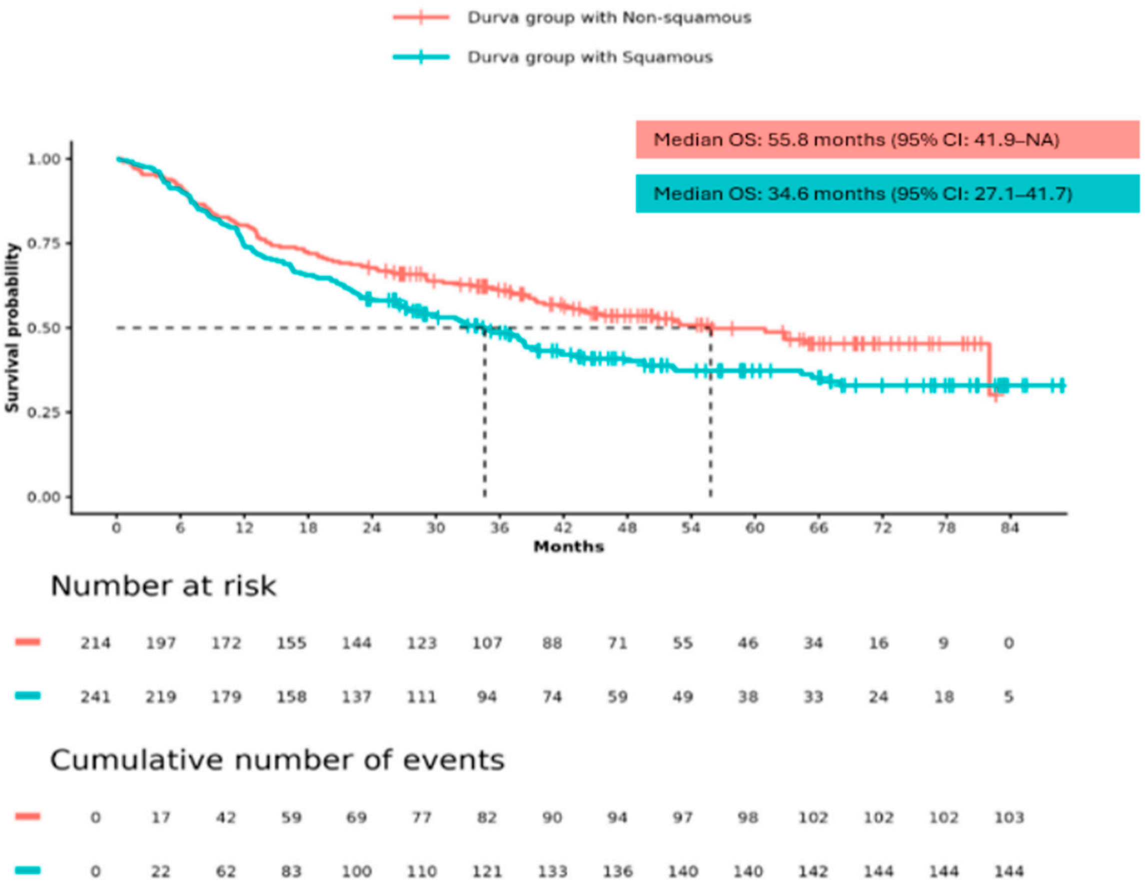

Durva: durvalumab

Figure S6. TDDM from cCRT initiation, stratified by PD-L1 status.

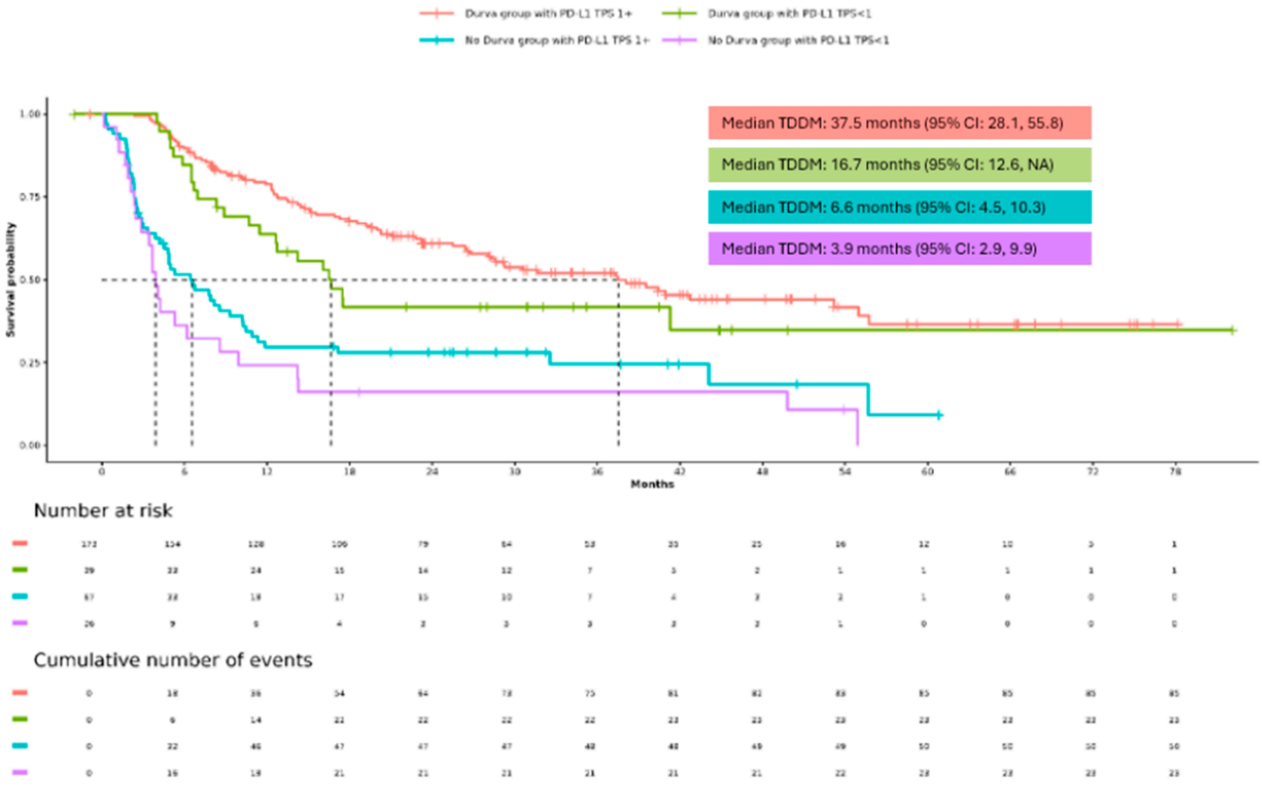

cCRT: Concurrent Chemoradiotherapy; Durva: durvalumab; PD-L1; Programmed Death Ligand 1; TDDM: Time to distant metastases or death

Table S3 Disease progression, stratified by PD-L1 status

Disease progression outcomes	cCRT + Durvalumab		cCRT-only	
	PD-L1 ≥1% (N=174)	PD-L1 <1% (N=40)	PD-L1 ≥1% (N=67)	PD-L1 <1% (N=26)
**Disease Progression***				
**Yes (n, %)**	64 (36.8%)	20 (50.0%)	35 (52.2%)	12 (46.2%)
**Disease progression type**				
**Locoregional**	5 (2.9%)	2 (5.0%)	0 (0.0%)	2 (7.7%)
**Distant metastasis**	46 (26.4%)	14 (35.0%)	28 (41.8%)	7 (26.9%)
**Initiated subsequent treatment**	13 (7.5%)	4 (10.0%)	7 (10.4%)	3 (11.5%)
**Time from cCRT initiation to distant metastasis (month)**				
**Mean (SD)**	14.6 (11.0)	8.81 (4.34)	8.05 (10.2)	10.4 (19.7)
**Median [Min, Max]**	12.5 [2.37, 53.2]	6.82 [4.01, 17.5]	5.93 [0.329, 55.7]	3.65 [0.131, 54.9]

*Based on the definition of disease progression, which included patients who presented evidence of progression (recurrence, metastasis, or patients who did not flag the presentation of disease progression but continued with subsequent treatment). cCRT: Concurrent chemoradiotherapy; PD-L1: Programmed Death Ligand; SD: Standard deviation; Min, Max: Minimum, Maximum.

Figure S7. First subsequent treatment following disease progression, stratified by PD-L1 status

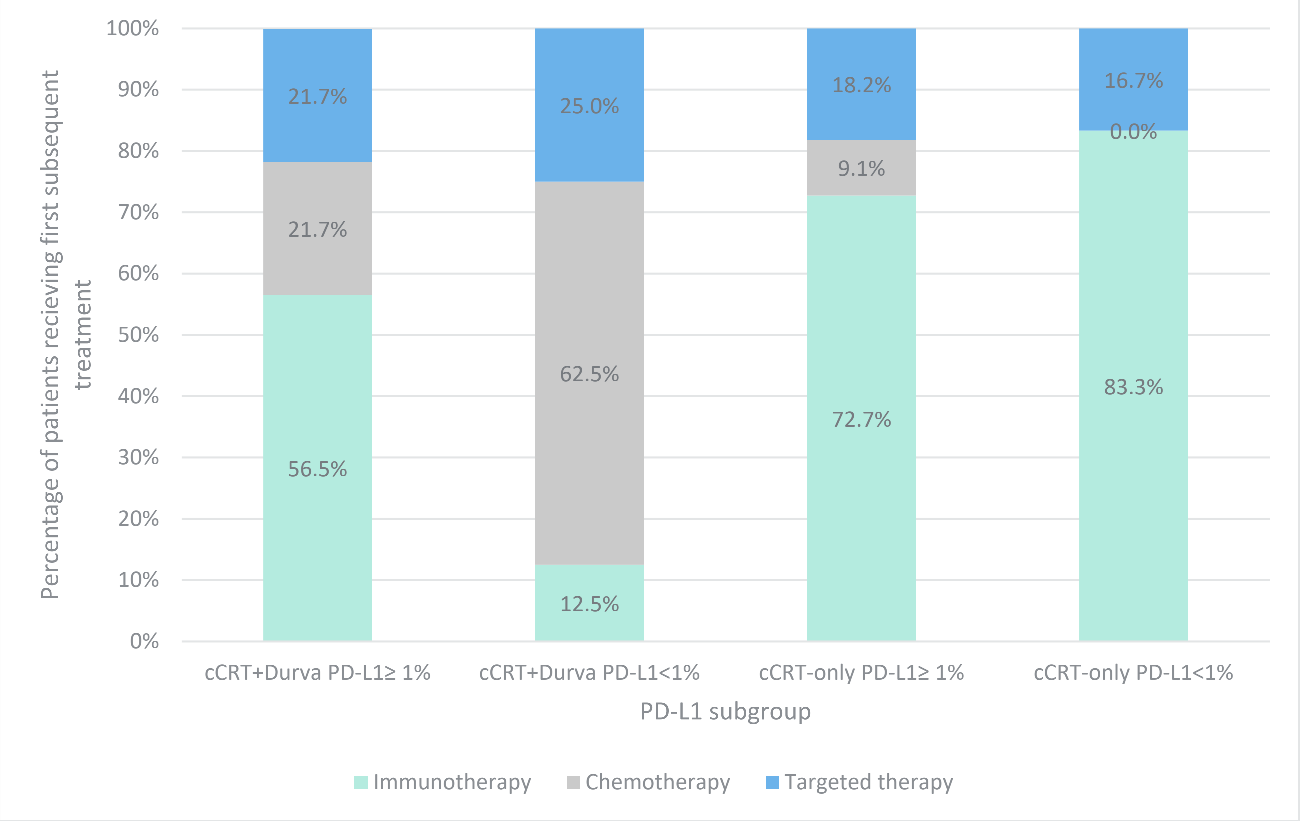

cCRT: Concurrent Chemoradiotherapy; Durva: durvalumab; PD-L1; Programmed Death Ligand 1

Figure S8. Overall survival from cCRT initiation, stratified by PD-L1 status.

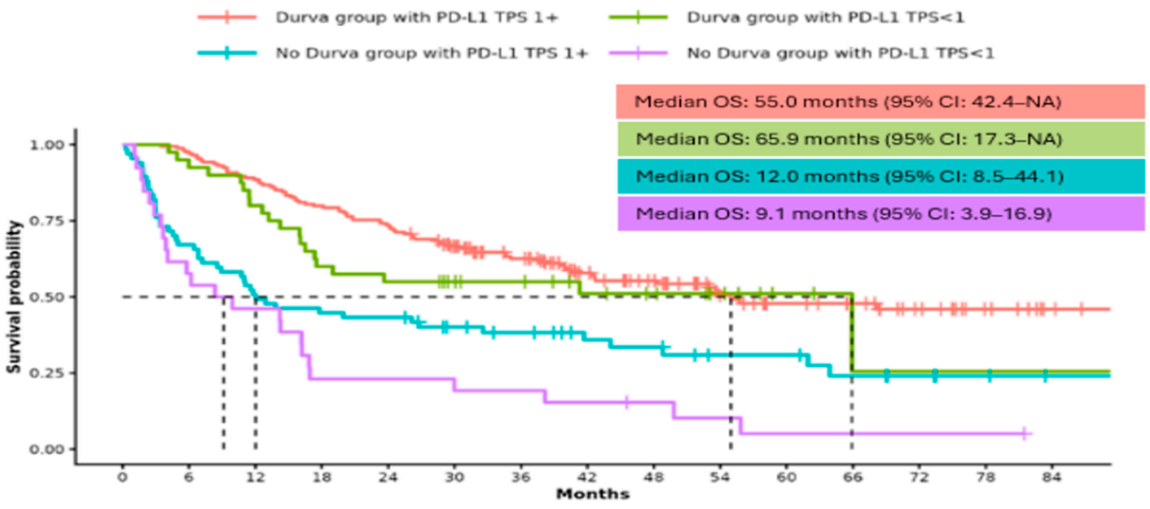

Number at risk

Durva group with PD-L1 TPS 1+	174	169	154	139	128	108	90	68	57	38	31	27	19	11	5
Durva group with PD-L1 TPS<1	40	37	32	24	22	19	17	13	11	7	3	1	1	1	1
No Durva group with PD-L1 TPS 1+	67	45	34	30	29	23	20	15	14	10	10	7	5	3	1
No Durva group with PD-L1 TPS<1	26	15	12	6	6	5	5	4	3	2	1	1	1	1	0

Cumulative number of events

Durva group with PD-L1 TPS 1+	0	5	20	35	46	58	64	70	73	77	79	79	80	80	80
Durva group with PD-L1 TPS<1	0	3	8	16	18	18	18	19	19	19	19	20	20	20	20
No Durva group with PD-L1 TPS 1+	0	22	33	37	38	40	41	42	43	44	44	46	46	46	46
No Durva group with PD-L1 TPS<1	0	11	14	20	20	21	21	22	22	23	24	24	24	24	24

cCRT: Concurrent chemoradiotherapy; Durva: durvalumab; PD-L1: Programmed death ligand 1; TPS: Tumor Proportion Score

Figure S9. Overall survival from durvalumab initiation, stratified by PD-L1 status.

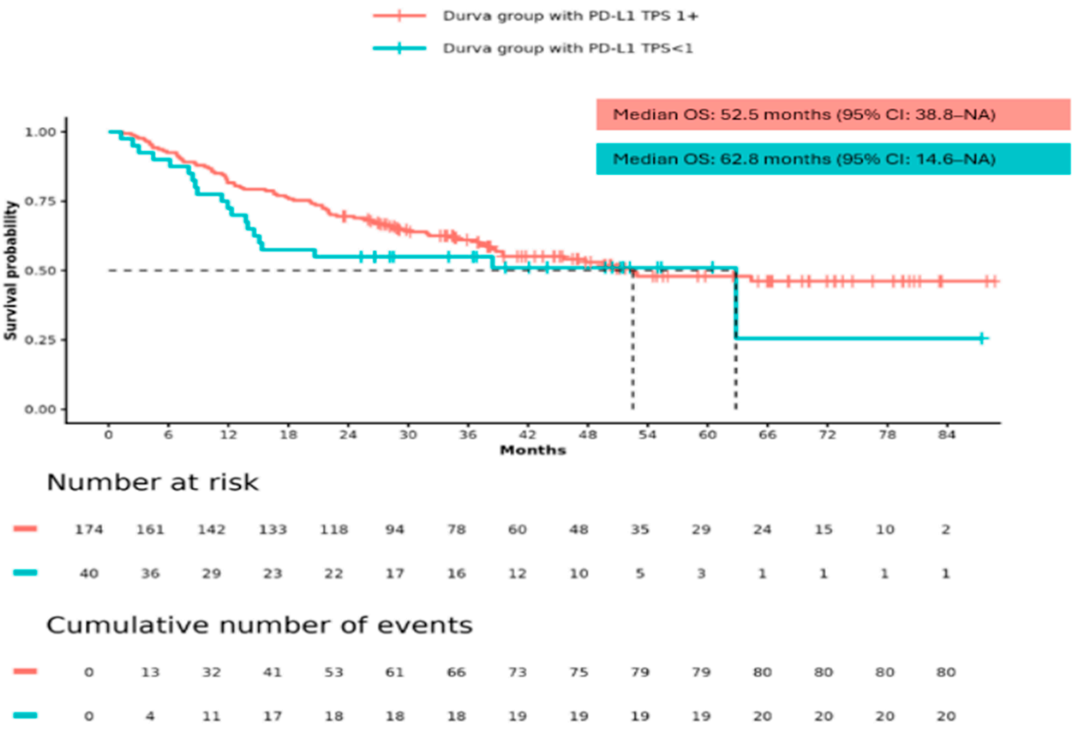

cCRT: Concurrent chemoradiotherapy; Durva: durvalumab; PD-L1: Programmed death ligand 1; TPS: Tumor Proportion Score
